# Bilateral Radial Artery Aneurysms Following Trauma: A Case Report

**DOI:** 10.7759/cureus.85148

**Published:** 2025-05-31

**Authors:** Rohit K Goru, Rakesh Shah, Shaun Cardozo

**Affiliations:** 1 Internal Medicine, Wayne State University School of Medicine, Detroit, USA; 2 Internal Medicine, Detroit Medical Center, Wayne State University, Detroit, USA; 3 Cardiology, Wayne State University, Detroit, USA

**Keywords:** end-to and anastomosis, extremity trauma, fall injury, radial artery aneurysm, true aneurysm

## Abstract

Radial artery aneurysms are rare and are usually secondary to either blunt or penetrating traumatic etiology, such as catheterizations via radial artery access, bone fractures, or occupational injuries. Besides trauma, radial artery aneurysms may be related to idiopathic, infectious (mycotic), atherosclerotic etiology, or connective tissue diseases such as Marfan syndrome. However, bilateral radial artery aneurysms are extremely rare. A 61-year-old male developed pulsatile masses on both wrists following syncopal episodes that led to falls. Imaging confirmed bilateral radial artery aneurysms. The patient underwent surgical resection of both aneurysms - first on the right wrist, followed by the left after cellulitis resolution. Her postoperative course was uneventful. Histopathological examination showed findings suggestive of the true aneurysm. This rare case underscores the importance of early diagnosis and surgical intervention for bilateral radial artery aneurysms, particularly following trauma.

## Introduction

Radial artery aneurysms are rare and are usually secondary to traumatic etiology, such as catheterizations via radial artery access, bone fractures, or occupational injuries [1]. Pseudoaneurysms are more commonly reported secondary to trauma than true aneurysms [2]. Besides trauma, radial artery aneurysms may be related to idiopathic, infectious (mycotic), atherosclerotic etiology, or connective tissue diseases such as Marfan syndrome. Bilateral radial artery aneurysms are extremely rare [3]. We report a unique case of bilateral radial artery aneurysms secondary to blunt trauma in a patient with recurrent syncope.

## Case presentation

A 61-year-old male presented after a syncopal episode that resulted in a fall, injuring his left wrist and resulting in a painful pulsatile mass. The patient also reported a syncopal episode one month prior that resulted in a similar lesion on his right wrist. The patient has a personal history of chronic hepatitis C, cerebral vascular accident (CVA) with residual right-sided weakness, IV heroin and cocaine use, and a remote history of seizure disorder well-controlled on phenytoin monotherapy. He had no personal or family history of aneurysmal disease.

On the exam, the patient had warm, pulsatile, extremely tender swelling over the radial aspect of bilateral wrists near the anatomical snuff box. No audible bruits or other vascular abnormalities were noted. Examination of the left wrist also suggested cellulitis. The physical exam showed normal stature and appropriate limb lengths and was negative for any stigmata of collagen vascular disease.

**Figure 1:**
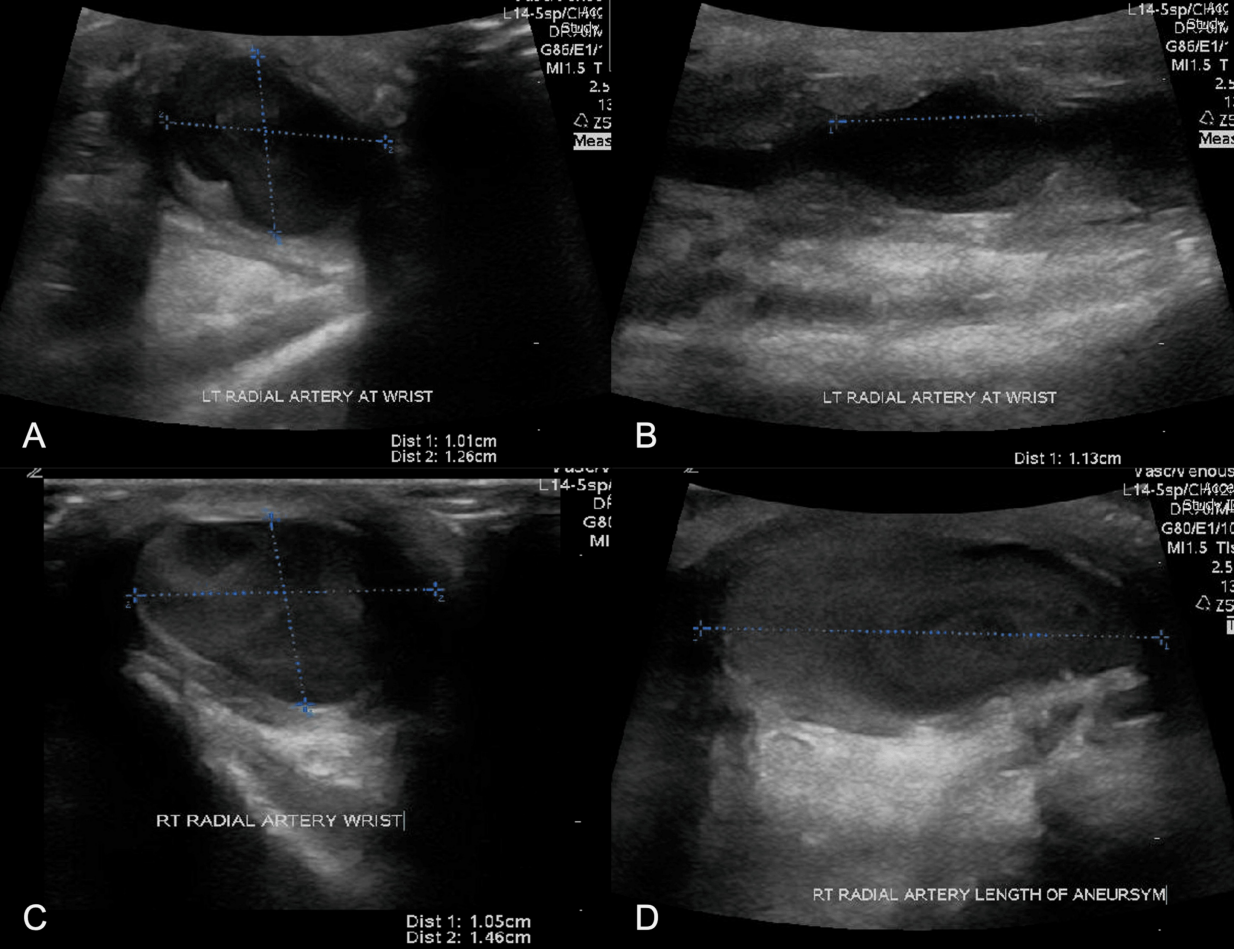
Doppler imaging of bilateral forearms. (A) Left wrist: Radial artery aneurysm measuring 1.01 cm in height and 1.26 cm in width. (B) Left wrist: Length of the radial artery aneurysm measuring 1.13 cm. (C) Right wrist: Radial artery aneurysm measuring 1.05 cm in height and 1.46 cm in width. (D) Right wrist: Length of the radial artery aneurysm measuring 2.57 cm.

Plain radiographs of the bilateral wrists showed no fracture or dislocation. Doppler ultrasonography demonstrated a 1.01 x 1.26 x 1.13 cm aneurysmal dilatation in the left radial artery (average radial artery diameter ranges between 2.0 and 3.0mm) (Figures 1A, 1B) and a 1.05 x 1.46 x 2.57 cm dilatation in the right radial artery (Figures 1C, 1D). Allen’s test demonstrated radial dominance of the palmar arch bilaterally without compromised blood flow. Duplex ultrasound showed no vascular abnormalities of the bilateral carotid arteries, lower extremities, and aorto-iliac system.

Exploration of the right radial artery with excision and ligation of the radial artery aneurysm was performed. Histopathological examination of the excised aneurysm of the right radial artery revealed the presence of all three layers of arterial wall, suggestive of a true aneurysm. It showed a thickened intima, fibro-myxoid degeneration of the media, and elastic tissue degeneration. Repair of the left radial aneurysm was deferred for one month for the cellulitis to be resolved. At that time, the patient underwent excision of the left radial artery aneurysm, with end-to-end anastomosis of the proximal and distal radial arteries using a saphenous vein graft. Histopathological analysis confirmed the presence of a true aneurysm, characterized by all three arterial wall layers with marked perivascular inflammatory cellular infiltrate, but no fibrinoid necrosis. Pathology interpretation was not diagnostic of vasculitis but instead more suggestive of nonspecific inflammation. The patient experienced symptomatic relief after the surgery and was discharged home.

Workup for syncope revealed no structural neurological abnormalities or epileptiform activity on electroencephalogram. Cardiac workup revealed non-obstructive coronary artery disease on left heart catheterization and no structural abnormalities on echocardiogram. An electrophysiological study was performed on a previous admission, and a loop recorder was placed to identify the etiology for his recurrent syncopal episodes. Interrogation of the loop recorder revealed three 30-second episodes of atrial flutter on the night of syncope, and the patient subsequently underwent successful atrial flutter ablation.

## Discussion

Our patient presumably developed bilateral radial artery aneurysms due to blunt trauma from multiple syncopal episodes leading to falls. This patient's history of IV drug use may have also contributed to further arterial wall fragility, possibly predisposing this patient to develop radial artery aneurysms [4]. Only the radial arteries were involved, with sparing of other vascular territories. There were no clinical symptoms, signs, or histopathological findings suggestive of atherosclerosis or connective tissue disease. This patient's radial aneurysms, resulting from trauma secondary to arrhythmogenic syncope, present an exceptionally rare clinical scenario.

True radial artery aneurysms, characterized by the involvement of all three layers of the arterial wall, are uncommon and typically result from significant trauma, repetitive stress, or iatrogenic injury. The literature first describes a case of radial artery aneurysm in 1966 by Thorrens et al. At that time, it was thought to be secondary to arteriosclerosis [5]. Subsequently, many cases of radial artery aneurysms have been reported in the literature [1]. Radial artery aneurysms constitute about 1% and 2.9% of all peripheral arterial and upper extremity arterial aneurysms, respectively [6]. Most radial artery aneurysms are pseudoaneurysms associated with penetrating or iatrogenic trauma, such as radial artery cannulation. True radial artery aneurysms are rare and are often associated with genetic predispositions such as Marfan syndrome or other genetic mutations in structural proteins such as platelet-derived growth factor-β [3,7]. Blunt trauma causing a true aneurysm is rarely reported and is more common in the ulnar artery than the radial artery [8]. Radial artery aneurysms are usually located distally, close to the anatomical snuffbox in the majority of cases, as seen in many cases reported in the literature [3,7,9-12]. Our case is unique given the involvement of both radial arteries secondary to trauma, which likely involved falling on an everted wrist.

Presentation can be varied depending on the size and extent of involvement. There are no specific guidelines for management, and it depends mostly on patient symptoms with distal vascular compromise, intra-aneurysmal thrombi, or associated infections. Aneurysms can be complicated by embolization and rupture. While the exact risk of these complications is not fully established, it is generally considered to be greater when a thrombus is present within the aneurysm sac or when the aneurysm’s diameter is more than 2.5 times that of the native artery [13]. Duplex ultrasound and computed tomography angiography (CTA) can assist in diagnosing the condition and help assess the size of the aneurysm.

Small asymptomatic aneurysms can be clinically observed for progression in size and worsening of symptoms. Management of symptomatic aneurysms involves excision of lesions with graft placement if needed. Allen’s test is also essential to determine the flow distal to radial or ulnar artery aneurysms since this helps determine acuity and severity, guiding further management. Our patient was presented with pain and an exquisitely tender aneurysm located close to an anatomical snuff box, and hence, it was managed with surgery.

## Conclusions

True distal radial artery aneurysms are a rare condition. If not diagnosed correctly, they can lead to serious complications, including thrombosis, embolization, rupture, and compression of adjacent structures. Physicians should be vigilant and consider this condition in their differential diagnoses, especially in the setting of trauma to the upper extremities. While there are no clear guidelines, surgery is generally the primary treatment in most cases.

## References

[1] Shaabi HI (2014). True idiopathic saccular aneurysm of the radial artery. J Surg Case Rep.

[2] Gabriel SA, Marcondes de Abreu MF, Gonçalves de Abreu GC, Cabrini Simões CR, Guedes Chrispim AC, de Camargo Júnior O (2013). True posttraumatic radial artery aneurysm. J Vasc Bras.

[3] Yukios U, Matsuno Y, Imaizumi M, Mori Y, Iwata H, Takiya H (2009). Bilateral radial artery aneurysms in the anatomical snuff box seen in marfan syndrome patient: case report and literature review. Ann Vasc Dis.

[4] Naqi SA, Khan HM, Akhtar S, Shah TA (2006). Femoral pseudoaneurysm in drug addicts - excision without revascularization is a viable option. Eur J Vasc Endovasc Surg.

[5] Thorrens S, Trippel OH, Bergan JJ (1966). Arteriosclerotic aneurysms of the hand. Excision and restoration of continuity. Arch Surg.

[6] Ogeng'o JA, Otieno B (2011). Aneurysms in the arteries of the upper extremity in a Kenyan population. Cardiovasc Pathol.

[7] Shalhub S, Hysa L, Byers PH, Meissner MH, Ferreira M Jr (2021). True radial artery aneurysm in a patient with somatic mosaicism for a mutation in platelet-derived growth factor receptor β gene. J Vasc Surg Cases Innov Tech.

[8] Clark ET, Mass DP, Bassiouny HS, Zarins CK, Gewertz BL (1991). True aneurysmal disease in the hand and upper extremity. Ann Vasc Surg.

[9] Al-Zoubi NA (2018). Idiopathic true aneurysm of distal radial artery: case report. Vasc Health Risk Manag.

[10] Ghaffarian AA, Brooke BS, Rawles J, Sarfati M (2018). Repair of a symptomatic true radial artery aneurysm at the anatomic snuff box with interposition great saphenous vein graft. J Vasc Surg Cases Innov Tech.

[11] Madeline Chee YM, Lew PS, Darryl Lim MJ (2020). True idiopathic radial artery aneurysm: a case report and review of current literature. EJVES Vasc Forum.

[12] Jawas A, Mohamed H, Almheiri M, Alshamsi S (2022). Snuff box radial artery aneurysm: a case report and literature review. Int J Surg Case Rep.

[13] Ayers J, Halbach J, Brown D (2015). True radial artery aneurysm: diagnosis and treatment. J Vasc Surg.

